# Assessment of Telomere Length in Archived Formalin-Fixed, Paraffinized Human Tissue Is Confounded by Chronological Age and Storage Duration

**DOI:** 10.1371/journal.pone.0161720

**Published:** 2016-09-06

**Authors:** Po-Lian Kong, Lai-Meng Looi, Tze-Pheng Lau, Phaik-Leng Cheah

**Affiliations:** Department of Pathology, Faculty of Medicine, University of Malaya, Kuala Lumpur, Federal Territory, Malaysia; University of Newcastle, UNITED KINGDOM

## Abstract

Telomeres shorten with physiological aging but undergo substantial restoration during cancer immortalization. Increasingly, cancer studies utilize the archive of formalin-fixed, paraffin-embedded (FFPE) tissues in diagnostic pathology departments. Conceptually, such studies would be confounded by physiological telomere attrition and loss of DNA integrity from prolonged tissue storage. Our study aimed to investigate these two confounding factors. 145 FFPE tissues of surgically-resected, non-diseased appendixes were retrieved from our pathology archive, from years 2008 to 2014. Cases from 2013 to 2014 were categorized by patient chronological age (0–20 years, 21–40 years, 41–60 years, > 60 years). Telomere lengths of age categories were depicted by telomere/chromosome 2 centromere intensity ratio (TCR) revealed by quantitative fluorescence *in situ* hybridization. Material from individuals aged 0–20 years from years 2013/2014, 2011/2012, 2009/2010, and 2008 were compared for storage effect. Telomere integrity was assessed by telomere fluorescence intensity (TFI). Epithelial TCRs (mean ± SD) for the respective age groups were 4.84 ± 2.08, 3.64 ± 1.21, 2.03 ± 0.37, and 1.93 ± 0.45, whereas corresponding stromal TCRs were 5.16 ± 2.55, 3.84 ± 1.36, 2.49 ± 1.20, and 2.93 ± 1.24. A trend of inverse correlation with age in both epithelial and stromal tissues is supported by r = -0.69, p < 0.001 and r = -0.42, p < 0.001 respectively. Epithelial TFIs (mean ± SD) of years 2013/2014, 2011/2012, 2009/2010 and 2008 were 852.60 ± 432.46, 353.04 ± 127.12, 209.24 ± 55.57 and 429.22 ± 188.75 respectively. Generally, TFIs reduced with storage duration (r = -0.42, p < 0.001). Our findings agree that age-related telomere attrition occurs in normal somatic tissues, and suggest that an age-based reference can be established for telomere studies on FFPE tissues. We also showed that FFPE tissues archived beyond 2 years are suboptimal for telomere analysis.

## Introduction

Telomeres are specialized DNA-protein complexes made of hexameric DNA tandem repeats (TTAGGG) which protectively cap the terminals of linear chromosomes to prevent end fusion and loss of genomic DNA [1]. Telomeres of normal somatic cells undergo attrition after each round of cell division due to incomplete replication of telomeres during DNA synthesis [2]. When shortening of telomeres reaches a critical length, the cell reaches its Hayflick limit when cell division is arrested and the cell undergoes senescence or apoptosis [3–5]. Hence, telomere shortening is associated with increasing age [6–10]. Unlike normal somatic cells, cancer cells are able to bypass the Hayflick limit through the activation of telomerase [11] or alternative telomere lengthening pathways [12, 13]. Immortalization, a hallmark of malignancy, occurs as cancer cells overcome the proliferative barriers of replicative senescence and crisis [14–16]. The roles of telomerase and telomere length assessment in cancer and other pathological disorders remain an actively researched field [17–22]. Emerging investigations on the association of telomeres and age-related disorders advocate that telomeres of neoplastic cells are significantly shorter than normal somatic cells despite the ability of neoplastic cells to restore telomeres [23]. Also, normal somatic cells that have undergone multiple cell divisions would exhibit shortened telomeres that may possibly be comparable to neoplastic cells. Since telomere length is a proposed biomarker for physiological aging, the influence of age should be taken into consideration in telomere-related cancer studies to provide a better insight into the relationship of telomere length and cancer [24, 25].

Formalin-fixation and paraffin-embedding are commonly applied in processing of tissues for diagnostic microscopy. Such formalin-fixed, paraffin-embedded (FFPE) tissues are invaluable archived resources for further histological study and retrospective analyses as they are clinical samples linked with patient characteristics and follow-up information [26]. However, the utility of FFPE specimens for molecular studies is relatively limited compared with fresh frozen tissues [27]. This is because nucleic acid and proteins in FFPE tissues may be degraded or chemically modified by the fixation process. Also it is conceivable that prolonged storage of the blocks may contribute to degradation of DNA in FFPE tissues [28–32]. Since telomeres are composed of hexameric DNA tandem repeats, loss of DNA integrity in FFPE tissues would affect telomere length measurements. Thus, in the utilization of FFPE tissues within diagnostic pathology departments for telomere studies, the contribution of storage duration should be considered.

Our study was conducted to gain insight into the significance of chronological age and storage duration of FFPE tissues on telomere length measurements using quantitative fluorescence *in situ* hybridization (Q-FISH). This proof of concept study aims to provide the justification and basis for a matrix study which would be important for biological studies on telomere losses and gains.

## Materials and Methods

### FFPE tissue retrieval

Study A: To investigate the influence of human age on telomere length, FFPE blocks of morphologically normal, non-diseased, surgically-excised human appendix tissues processed in years 2013 and 2014 were retrieved from the archives of the Department of Pathology, University of Malaya Medical Centre (UMMC). These tissue samples were categorized into 4 groups according to age of the patients: 0–20 years, 21–40 years, 41–60 years, and > 60 years.

Study B: To investigate the influence of storage duration on telomere length, FFPE blocks of morphologically normal, non-diseased, surgically-excised human appendix tissues from individuals aged 0–20 years were retrieved from the pathology collection of years 2008 to 2014. These FFPE tissues were categorized into 4 groups, according to the years when surgery were performed: 2013/2014, 2011/2012, 2009/2010, and 2008.

Positive control: Two FFPE blocks of surgically-excised normal testicular tissue processed in year 2014, one each from two individuals aged 63 years and 76 years served as positive controls for telomere detection. Both were removed as part of the treatment for prostate cancers and were confirmed to be histologically normal testicular tissues. It is well-established that the telomere length equilibrium is maintained in germ cells, and thus, spermatogonia and spermatocytes in testicular tissues [33] would provide sufficient length of telomeres to serve as positive controls for successful telomere length measurement with our Q-FISH protocol. For each telomere Q-FISH run, a FFPE testis tissue section was included.

Hematoxylin and eosin (H&E) stained histological sections from the FFPE blocks were examined prior to inclusion into the study. For inclusion into studies A and B, the tissue had to exhibit good preservation of the mucosa. Appendixes that were inflamed or showed the presence of any specific pathology (e.g. neoplasia, cysts, fibrosis, atresia, parasites) were excluded from the study. The Q-FISH procedure for both studies were undertaken between December 2014 and March 2015. All cases in the 2013/2014 batch were stained and imaged within 2 years of storage duration.

### Ethics statement

All the patients provided informed and written consent to undergo the surgical procedures through which the appendix was removed. However, because this was a study on archived tissue (pathology repository) accessed retrospectively, it was not possible to contact and seek consent from the patients a second time for the purpose of this research. The Medical Research Ethics Committee/Institutional Review Board (IRB) of this Medical Centre accepts that for studies on archived excision and biopsy material which are retrospective and observational in nature, additional consent from the patients are not required. However, patient personal information and identity has to be anonymized, data confidentiality maintained, the study data handled only by the research team; and the study conducted in compliance with the Declaration of Helsinki. The IRB has reviewed this study protocol and granted approval for the study (reference number 1182.2).

### Marking the region of interest (ROI)

H&E stained slides of recruited FFPE blocks were examined by the study pathologist (LML) to identify areas of the mucosa as the region of interest (ROI) for telomere Q-FISH analysis. The ROI targeted differentiated epithelial cells from appendiceal glands, avoiding the lower crypts and basal layer which are the source of proliferating cells. A four-μm thick tissue section was then cut from each FFPE tissue block and mounted on positively-charged glass slides. Based on visual comparison with the corresponding H&E slide, the ROI was marked out with a diamond pen.

For Q-FISH analysis, imaged epithelial cells from the ROI were visually selected from the luminal aspect of the glands, i.e. cells showing cytoplasmic mucinous vacuoles (e.g. goblet cell morphology) as a marker of differentiation. Stromal cells were selected based on morphology and location, these being cells with oval nuclei adjacent to the glandular epithelium but separated from the epithelium by a basement membrane. Small, round cells that could be lymphocytes, as well as endothelial cells (surrounding vascular spaces) were excluded.

### Deparaffinization, pretreatment, and enzymatic digestion

The sections were allowed to dry at room temperature and then baked at 68°C for 10 min. Next, they were deparaffinized twice, for 5 min each, in xylene, and then hydrated through a series of ethanol of decreasing concentrations (i.e. 100%, 96%, 70%) followed by deionized water (twice), for 2 min each. The slides were then incubated in Tris-EDTA buffer (10 mM Tris base, 1 mM EDTA solution, 0.05% Tween-20, pH 9.0) at 80°C for 30 min, then allowed to cool down at room temperature for 20 min, and washed twice for 3 min each with deionized water. The tissue sections were then digested with 8 mg/ml pepsin (S3002, Dako, Denmark) in 0.01N HCl (pH 2.0) at 37°C for 15 min, and followed by two washing steps of 3 min each in deionized water. Subsequently, the tissues were dehydrated with 96% ethanol, and allowed to dry at room temperature.

### Telomere Q-FISH

The commercially available Telomere Peptide Nucleic Acid (PNA) FISH Kit/FITC (K5325, Dako, Denmark) was used to perform telomere Q-FISH. Cy3-labelled centromere of human chromosome 2 (CEN2)-specific PNA probe (Dako, Denmark) was mixed with the FITC-labelled telomere-specific PNA probe (1:10) to make up the working solution. 2 μl of the mixture was applied on each slide where the ROI was marked. The slides were covered with 10 × 10 mm coverslips and sealed with rubber cement. Next, the targets and probes were denatured at 80°C for 5 min, and incubated in the dark at 37°C for 18 hours for hybridization. The slides were then rinsed briefly in 2 x SSC + 0.1% Tween-20 buffer (pH 7.0–7.5), and washed twice for 10 min each with the same solution at 58°C, followed by another wash at room temperature for 1 min. The slides were dehydrated with a series of increasing concentrations of ethanol (i.e. 70%, 85%, 96%) before being allowed to air-dry. Vectashield mounting medium containing 1.5 μg/ml DAPI nuclear counterstain (H-1200, Vector Laboratories, USA) was diluted with Vectashield mounting medium (H-1000, Vector Laboratories, USA) to produce a mixture of mounting medium containing 0.05 μg/ml DAPI. 5 μl of the mixture was added on each slide where the ROI was marked. The slides were then covered with 18 × 18 mm coverslips, and incubated in the dark at room temperature for 15 min before image analysis.

### Image capturing and telomere length analysis

Imaging of the slides was done as soon as possible after Q-FISH staining, but no later than 48 hours after [34]. The slides were viewed under a Zeiss Axio Imager Z2 epi-fluorescence microscope (Carl Zeiss, Germany) coupled with a CoolCube 1 charge-coupled device camera (Metasystems, Germany). Image acquisition and analysis were performed using the MetaCyte Lite software ver. 3.11.3 (Metasystems, Germany). The FITC, Cy3, and DAPI microscopical images were captured under x100 oil objective.

Study A: For each case, 30 non-overlapping epithelial interphase nuclei and 30 non-overlapping mucosal stromal interphase nuclei were selected within the ROI for the quantification of telomere length. The identification of nuclei was based on the colour composite images, and only epithelial and stromal cells with two CEN2 signals (Cy3) were included in the analysis. Telomere (FITC) and CEN2 (Cy3) signals of each nucleus were expressed as fluorescence intensities after correcting for background autofluorescence. The telomere length of each nucleus was determined by calculating the signal intensity of telomere to CEN2 ratio (TCR). The average TCR of 30 nuclei represented the estimated telomere length quantified for epithelial or stromal cells of each case. TCR was used for assessment in study A to counter variations contributed by sample and experimental conditions, as both centromeric and telomere signals of each case would be equally affected by those conditions.

Study B: For each case, 30 non-overlapping epithelial interphase nuclei were selected within the ROI, based on criteria described earlier, for the quantification of telomere length. The identification of nuclei was based on the colour composite images. Telomere fluorescence intensity (TFI) was used to represent telomere length to study the influence of storage duration of FFPE materials on telomere integrity, since the study was specifically looking into the measurability of telomere signals. Since CEN2 fluorescence signals were also captured by the software, their intensities were also compared against storage duration, although CEN2 was not the objective of this study.

### Statistical analysis

One-way analysis of variance (ANOVA) with Tukey’s honest significant difference (HSD) *post hoc* test was used to evaluate telomere length differences between age groups and differences of telomere integrity between years of surgery. Pearson’s correlation coefficient was used to correlate and determine the relationship of telomere length and chronological age, as well as telomere integrity and years of FFPE blocks storage duration after surgery. For all statistical outcomes, a p-value < 0.05 was considered significant. All statistical analyses were performed with SPSS version 22.0 for Windows (IBM, USA).

## Results

Study A: Seventy (70) FFPE blocks were recruited into the study on influence of human age on telomere length. These were appendixes removed during surgery but found to have no pathology histologically. The reasons for surgery were: suspected acute appendicitis, Hirschsprung’s disease, Meckel’s diverticulum, intestinal malrotation, intestinal obstruction, pyelonephritis, peritonitis, hepatic flexure tumor, intestinal hernia, annular pancreas, ovarian cyst, and ovarian or colorectal tumor. These comprised 18 from patient age group 0–20 years, 33 from 21–40 years, 10 from 41–60 years, and 9 from patients > 60 years old. All the recruited FFPE appendix tissues had been evaluated and confirmed by the study pathologist (LML) to be morphologically and histologically normal, and without specific pathology.

Study B: Ninety-three (93) FFPE blocks were recruited for the study on influence of storage duration on telomere length. As in study A, these were appendixes removed during surgery but found to have no pathology histologically. The reasons for surgery were: suspected acute appendicitis, enteric duplication cyst, ovarian cyst, benign mesenteric cyst, lymphangioma, benign mesenchymal lesion, Meckel’s diverticulum, Hirschsprung’s disease, and chronic cholecystitis. Of these, 18 were from years of surgery 2013/2014, 30 from 2011/2012, 22 from 2009/2010, and 23 from 2008. All the recruited FFPE appendix tissues had been evaluated and confirmed by the study pathologist (LML) to be morphologically and histologically normal, and without specific pathology.

**Fig 1:**
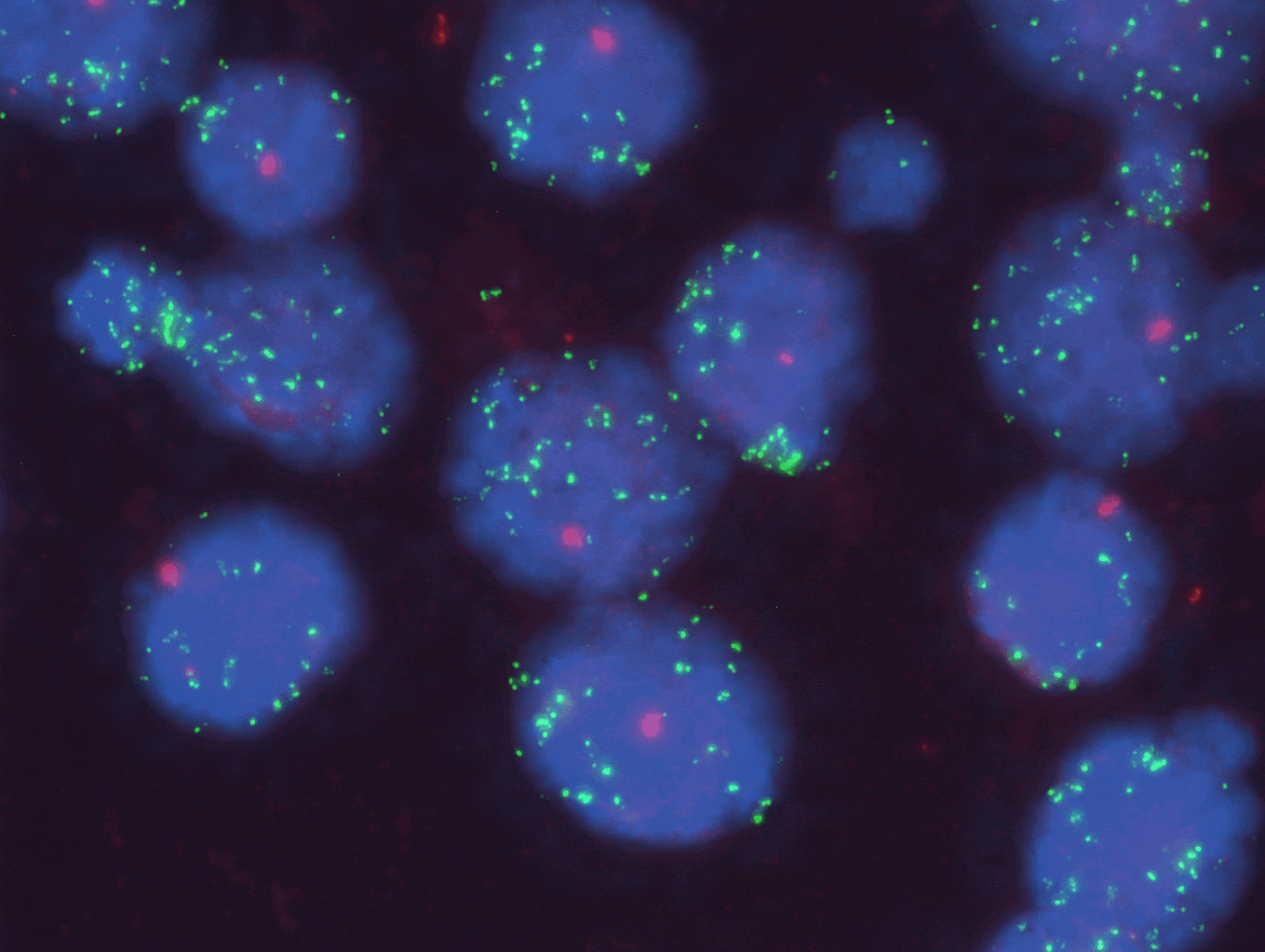
Fluorescence image of telomere Q-FISH denoting spermatocytes from normal testicular tissue of a 63-year-old individual used as experimental positive control. Q-FISH performed with PNA FITC-labelled telomere probe (green) and Cy3-labelled CEN2 probe (red), and nuclei counterstained with DAPI (blue) (original magnification: ×1000). The telomere signals (green) were very strong in the germ cells.

Positive control: A section of either control was included with every Q-FISH test run. It is noteworthy that spermatocytes, being haploid, expressed only a single CEN2 signal (red) per cell and this was observed in both testicular controls. In addition, 30 spermatocyte nuclei were analyzed for each testicular control. We found the telomere signals (green) to be extremely bright (Fig 1), yielding average TCR and TFI values (mean ± SD) of 8.98 ± 2.91 and 2406.97 ± 571.71 for the first control (patient’s age: 63 years), and 12.70 ± 2.57 and 2985.33 ± 1008.10 for the second control (patient’s age: 76 years).

### Study A: Influence of chronological age on telomere length

**Fig 2:**
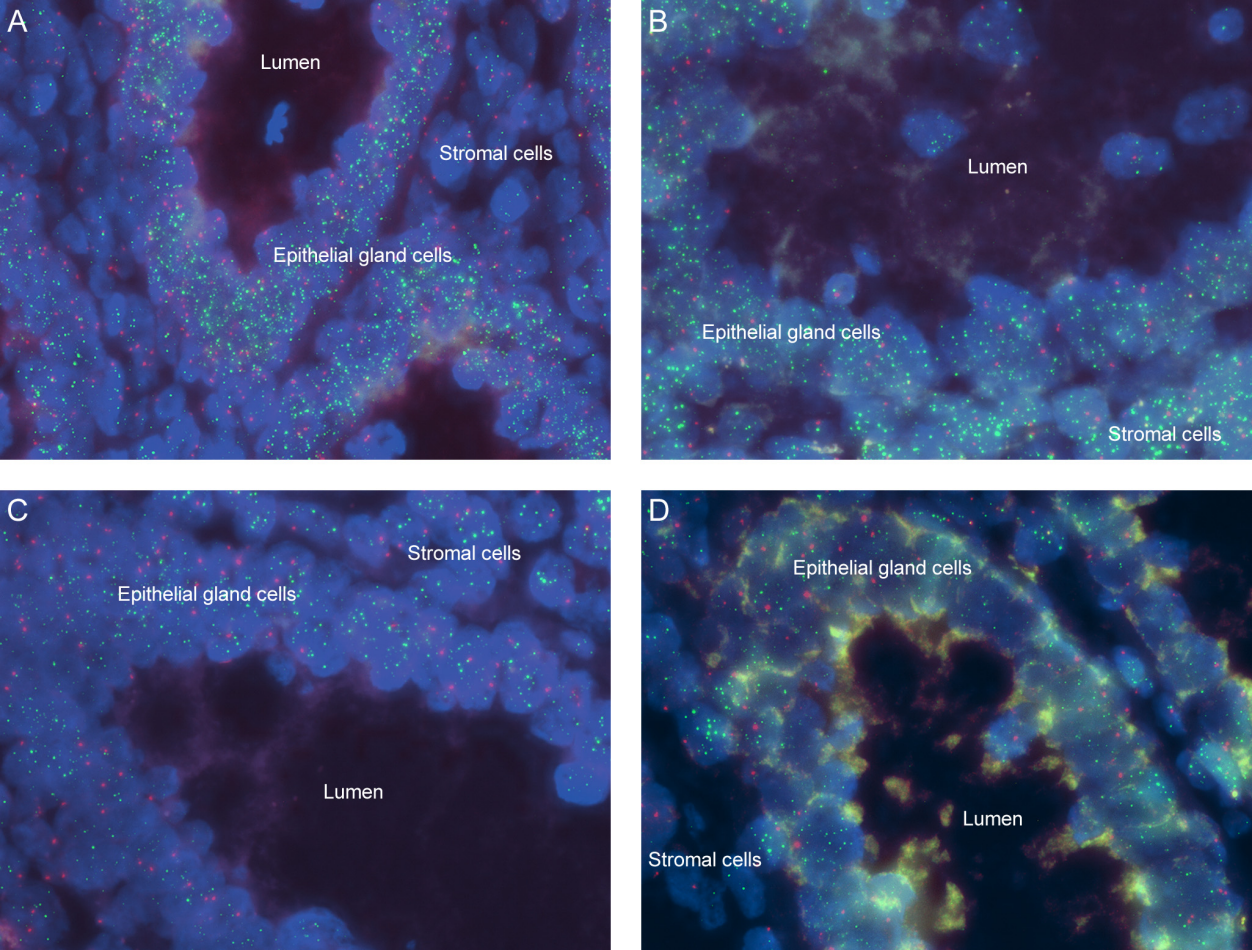
Representative fluorescence images of telomere Q-FISH from various patient age groups. Q-FISH performed with PNA FITC-labelled telomere probe (green) and Cy3-labelled CEN2 probe (red), and nuclei counterstained with DAPI (blue) (original magnification: ×1000). (A) Strong telomere signals (green) are evident in the normal appendix epithelial cells of a 7-week-old infant (age group: 0–20 years). (B) Bright telomere signals are observed in the normal appendix epithelial cells of a 22-year-old individual (age group: 21–40 years), but are slightly reduced in comparison with A. (C) Moderately bright telomere signals in the normal appendix epithelial cells of a 43-year-old individual (age group: 41–60 years), but are reduced compared to younger individuals. (D) Diminished telomere signals in the normal appendix epithelial cells of a 64-year-old individual (age group: > 60 years), compared to those of the younger individuals.

**Fig 3:**
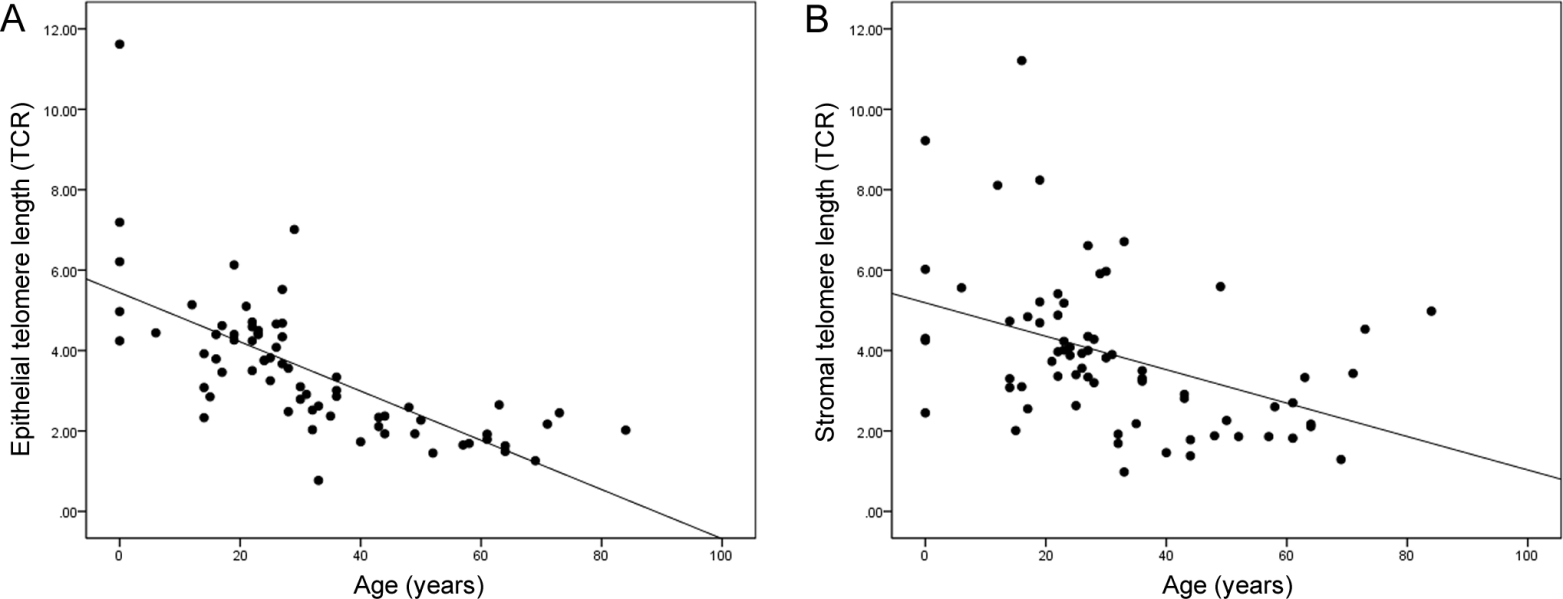
Scatter plots showing the inverse relationship between telomere length and chronological age. (A) Epithelial telomere length decreases with increasing chronological age. (B) Mucosal stromal telomere length decreases with increasing chronological age.

The TCRs (mean ± SD) of epithelial and mucosal stromal cells of the 70 normal appendixes archived in years 2013/2014 and categorized by patients’ age are summarized in Table 1. For both epithelial and stromal cells, telomere Q-FISH signals were the strongest in individuals of 0–20 years old, and diminished with age (Fig 2). Epithelial TCR differences between age groups were statistically significant, as revealed by one-way ANOVA (p < 0.001). Tukey’s HSD *post hoc* test shows that mean epithelial TCRs were significantly different between the 0–20 years group and those in categories 21–40 years, 41–60 years and > 60 years (p < 0.05). In addition, telomere lengths of those aged between 21–40 years old were significantly longer than those of 41–60 years and > 60 years (p < 0.05). There was however no significant epithelial TCR difference between age groups 41–60 years and > 60 years (Table 1). A strong, inverse correlation was also observed between epithelial telomere length and age (Pearson’s correlation coefficient r = -0.69, p < 0.001), supporting the concept of telomere shortening with increasing human age (Fig 3A).

**Table 1:** Telomere length (TCR) differences between age groups.

		Epithelial cells	Stromal cells
Age group (years)	n	TCR (Mean ± SD)	TCR (Mean ± SD)
0–20	18	4.84 ± 2.08	5.16 ± 2.55
21–40	33	3.64 ± 1.21	3.84 ± 1.36
41–60	10	2.03 ± 0.37	2.49 ± 1.20
> 60	9	1.93 ± 0.45	2.93 ± 1.24

Tukey’s HSD *post hoc* test. Epithelial cells: 0–20 vs. 21–40 years, p = 0.020; 0–20 vs. 41–60 years, p = 1.232 × 10^−5^; 0–20 vs. > 60 years, p = 1.203 × 10^−5^; 21–40 vs. 41–60 years, p = 0.009; 21–40 vs. > 60 years, p = 0.008; 41–60 vs. > 60 years, p = 0.998. Stromal cells: 0–20 vs. 21–40 years, p = 0.051; 0–20 vs. 41–60 years, p = 0.001; 0–20 vs. > 60 years, p = 0.012; 21–40 vs. 41–60 years, p = 0.143; 21–40 vs. > 60 years, p = 0.500; 41–60 vs. > 60 years, p = 0.946.

Similar to the findings in epithelial cells, one-way ANOVA analysis showed that mean stromal TCR differences between age-categories were statistically significant (p < 0.001). Tukey’s HSD *post hoc* test shows that the mean stromal TCR from individuals aged between 0–20 years (youngest age group) were significantly higher than those from the older age groups of 41–60 years and > 60 years, with p-values of 0.001 and 0.012, respectively. However, stromal TCR differences were not significant (p > 0.05) between the younger age groups of 0–20 years and 21–40 years, and between the older age groups of 41–60 years and > 60 years. Pearson’s correlation coefficient revealed an inverse relationship between stromal telomere length and age (r = -0.42, p < 0.001) (Fig 3B).

### Study B: Influence of duration of tissue storage on telomere length

**Fig 4:**
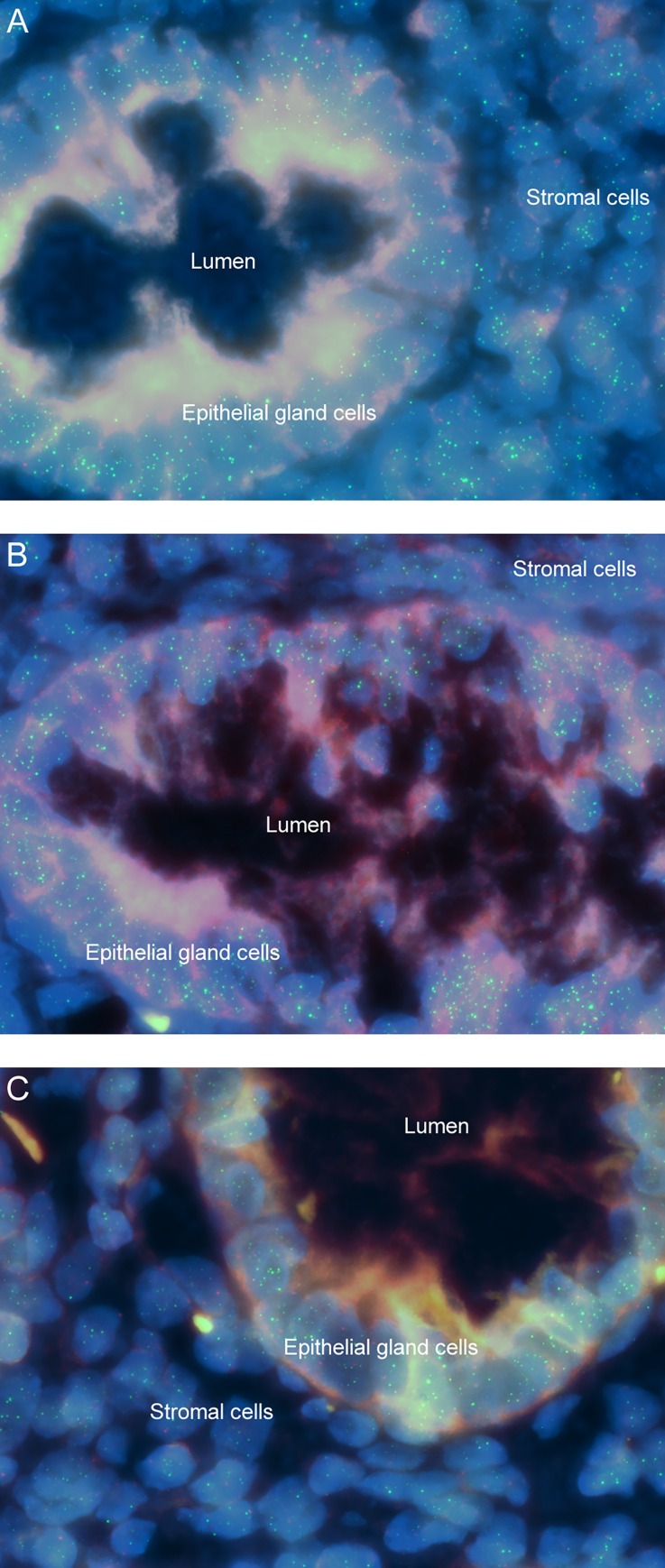
Fluorescence images of telomere Q-FISH from 0 to 20-year-old individuals after various storage durations. There are reduced telomere and CEN2 signals in the normal appendix epithelial cells from years (A) 2011/2012, (B) 2009/2010, and (C) 2008 compared to those from the most recent years (2013/2014) as depicted in Fig 2A.

For this study telomere length was represented by TFI as TCR is not suitable for telomere integrity assessment. Table 2 shows that there was significant difference between mean epithelial TFI measurements in appendixes removed in 2013/2014 (stored for ≤ 2 years before testing) compared with those removed in 2011/2012, 2009/2010, and 2008 (p < 0.001, one-way ANOVA with Tukey’s HSD *post hoc* test). TFI of appendixes archived in 2011/2012 did not differ from those archived in 2009/2010 and 2008 (p > 0.05). However, average TFI of appendixes archived in 2008 was significantly higher than those archived in 2009/2010 (p = 0.008). Fig 4 illustrates the telomere Q-FISH images of appendix tissues from years of surgery (A) 2011/2012, (B) 2010/2009, and (C) 2008 showing reduced telomere and CEN2 intensities in cells compared to Fig 2A. In general, an inverse correlation was observed between telomere length and years of surgery (Pearson’s correlation coefficient r = -0.42, p < 0.001), indicating that telomere integrity reduces with increased storage duration. Table 3 summarizes the findings with regards to CEN2 signals over storage duration. Notably, all tissues removed in 2013/2014 exhibited bright and quantifiable CEN2 signals, but only 2 samples from 2009/2010 had quantifiable signals. For tissues removed in 2011/2012 and 2008, none had quantifiable CEN2 signals.

**Table 2:** Mean telomere length (TFI) measurements in appendiceal epithelial cells according to storage duration.

Years of surgery (storage duration)	n	TFI (Mean ± SD)
2013/2014 (most recent, i.e. stored ≤ 2 years)	18	852.60 ± 432.46
2011/2012 (stored for > 2 to ≤ 4 years)	30	353.04 ± 127.12
2009/2010 (stored for > 4 to ≤ 6 years)	22	209.24 ± 55.57
2008 (stored for > 6 years)	23	429.22 ± 188.75

Tukey’s HSD *post hoc* test. 2013/2014 vs. 2011/2012, p = 3.379 × 10^−10^; 2013/2014 vs. 2009/2010, p = 7.615 × 10^−13^; 2013/2014 vs. 2008, p = 2.653 × 10^−7^; 2011/2012 vs. 2009/2010 years, p = 0.111; 2011/2012 vs. 2008, p = 0.614; 2009/2010 vs. 2008, p = 0.008.

**Table 3:** Number of samples with quantifiable CEN2 signals for each surgery year category.

Years of surgery (storage duration)	n	Number of samples with quantifiable CEN2 signals
2013/2014 (most recent, i.e. stored ≤ 2 years)	18	18
2011/2012 (stored > 2 to ≤ 4 years)	30	0
2009/2010 (stored for > 4 to ≤ 6 years)	22	2
2008 (stored for > 6 years)	23	0

## Discussion

Archived FFPE tissues is a rich repository that can be harnessed for many types of research studies. In the choice of a method for assessment of telomere length that can be applied to FFPE tissues, we decided to use Q-FISH as it allowed visualization of actual cells, which is the substrate of FFPE tissue sections. The ability to select specific cells for analysis, such as epithelial and stromal cells, as well as avoid cells (such as lymphocytes and endothelial cells), as shown in our study, offers attractive research potentials. Other techniques for telomere length measurement such as terminal restriction fragment analysis, polymerase chain reaction (PCR)-based techniques (e.g. quantitative PCR, monochrome multiplex PCR, absolute telomere length quantitative PCR) and single telomere length analysis are less appropriate as these techniques would require extraction of DNA from the FFPE tissues. As a common problem with FFPE-derived DNA is loss of DNA integrity, these techniques would have to contend with substantial bias from degraded or poor-quality DNA [35]. Moreover, telomere length measurement using these techniques would have included DNA from a mixture of different cells, introducing additional bias from stem cells and lymphocytes which have telomere lengths substantially different (longer) from the specific cells that one may wish to study, in contrast to the relative specificity achievable with Q-FISH [36].

In study A, TCR is used to represent telomere length for chronological age group telomere analysis. This is because the CEN2-specific PNA probe serves as an internal control to monitor for PNA probe hybridization efficiency in each tissue section, as CEN2 is not expected to be influenced by events that are correlated with telomere attrition, e.g. aging. Thus, the CEN2 signal intensity is used for normalization of fluorescence intensities, resulting in the TCR that is a surrogate for telomere length [37]. We have selected to use FFPE of non-diseased appendix tissues for this study because appendixes from a wide range of chronological age are available in our pathology archive, which is based on a large tertiary multidisciplinary hospital with a full range of paediatric and adult services. Recognizing that various disease conditions may affect the telomere length status of the appendix, we attempted to strictly control this aspect by recruiting only non-diseased and histomorphologically normal appendix in this study. Nevertheless, conditions that are not morphologically manifested and genetic variables have not been controlled, and this remains a limitation that should be considered in the interpretation of this study’s findings.

Although evidence point to telomere shortening occurring in epithelial cells more than in surrounding stromal cells [38, 39], we have included both glandular epithelial cells and mucosal stromal cells in this study to test the concept of telomere shortening with chronological age over two tissue types. The advantage of studying epithelial and stromal cells from the same tissue blocks is that they are controlled for technical variables such as collection, fixation, storage conditions, as well as the Q-FISH procedure. We studied telomere lengths of normal appendix epithelial and stromal cells across 4 different age groups, i.e. 0–20, 21–40, 41–60, and > 60 years. A decreasing trend in telomere length with increasing chronological age was shown in both epithelial and stromal cells. This is consistent with previous observations that human telomeres shorten with advancing age, indicating telomere length as a valid biomarker for aging [6, 8, 40–43]. Our study is unique because of the inclusion of a substantial proportion of individuals at a very young age (e.g. infants), compared with most other studies that focused on the older age groups. The latter scenario is probably because most tissue repositories would usually be stocked with material from pathological conditions such as cancers which usually afflict the middle-aged or elderly. Closer examination of our results shows that there was no significant difference in telomere length between individuals of the older age groups (i.e. 41–60 and > 60 years), and that both these groups possess significantly shorter telomeres than those from the youngest age group (0–20 years). This finding supports previous observation that telomeres of young individuals shorten rapidly, whereas once senescence sets in, the telomeres may not shorten much further [44].

Although our study demonstrated an inverse correlation of telomere length and chronological age in both epithelial and stromal tissues, our findings also caution that telomere lengths vary between different tissue types of the same individual [45, 46]. In our study, telomere lengths were, in general, longer in the stromal cells compared to epithelial cells (Table 1). Also the inverse correlation of stromal telomere length with age tapers off at an earlier age.

The notion of using normal, non-diseased tissue to determine telomere length as an age-baseline for the assessment of disease states is an attractive one with scientific merit. The relevance of normal age-baseline measurement is the prediction of the approximate telomere length of an individual at a certain age-range, providing an important reference for comparison against telomere lengths of diseased tissues in that individual. For mature individuals with already shorter telomeres due to physiological aging, there may be only minor differences in further telomere shortening attributable to disease. The differences could be better highlighted by a comparison with the telomere lengths measured from normal tissues as a reference point, thus providing better insights into the role of telomeres in disease states. Telomeres of some diseases such as cancer and hereditary premature aging may be significantly shorter than normal as a result of genetic mutations [47–50]. Because telomeres of young individuals are longer, shortening of telomeres due to disease may not be easily appreciated. Comparison against expected age-baseline measurements (reference range) can provide a “marker” to indicate the abnormality. However, because telomere lengths do vary from tissues to tissues, it would be unsafe to generalize findings across tissues and organs. Hence development of age-baselines should be tissue specific.

The second part of this study (study B) suggests that telomere integrity of FFPE tissues reduces over time. Our study shows that the average TFIs of FFPE appendix tissues from years of surgery 2011/2012 (stored for > 2 to ≤ 4 years), 2009/2010 (stored for > 4 to ≤ 6 years), and 2008 (stored for > 6 years) were significantly lower than those of years 2013/2014 (most recent FFPE tissues) of the corresponding age group. To date, this is the only study to demonstrate the effect of storage duration on the assessment of telomere length in FFPE tissues. Earlier studies have shown that DNA can be extracted from FFPE tissues that were archived several years to decades, but most of them were fragmented as many short sequences were detected after amplification with PCR using short primers [29, 51, 52]. We suggest that the reduction of telomere signals in FFPE tissues is due to DNA degradation, and also DNA fragmentation (breakdown of DNA into shorter fragments) leading to the loss of DNA segments [53]. This prevents the fluorescent probe from detecting specific sites on chromosomes as the targeted complimentary DNA is broken, reducing hybridization efficiency. This is also seen in the diminished signals of CEN2, an internal control used in this study to monitor probe hybridization efficiency. Our results also show that TCR can only be used to represent telomere length of the most recent FFPE tissues (years of surgery: 2013/2014), but may not produce reliable results using tissues stored for 2 years and longer as the diminished fluorescent signals will confound telomere length quantification. Similar to our observations, Hammond et al. found that the most recent bone marrow smear samples had the highest hybridization efficiency with the FISH method to detect centromeres of chromosomes X, 6 and 18, whereas the lowest was for the oldest samples [54]. We note that the average TFI value of tissues from year of surgery 2008 (stored for > 6 years) was significantly higher than those of years of surgery 2009/2010 (stored for > 4 to ≤ 6 years), although it was nevertheless much lower than those of years of surgery 2013/2014 (most recent, i.e. stored for ≤ 2 years). 10 (43.5%) of 23 individuals from surgery year 2008 were infants (0–2 years old). As infants have long telomeres, they would have contributed to the higher average TFI for that group. By way of comparison, for the years of surgery 2009/2010, and 2011/2012, 22.7% and 46.7% (respectively) of tissues were from infants.

The deterioration of telomere length measurability after such a short duration of 2 years is of concern. Before attributing this phenomenon to storage duration, the contribution of other variables which can affect telomere lengths should always be considered. In our medical complex (to which the pathology department belongs) procedures for fixation, collection and transportation of excision and biopsy tissues have been standardized for many years. Transportation is within the same building complex. Also, fixative solutions (buffered formalin) are prepared and issued by the Pathology Department to the operating suites of the medical complex. Hence, there is justification for considering the storage condition of the tissue blocks to be a major variable. In tropical countries such as Malaysia, where ambient room temperatures can range from 24°C to 30°C, deterioration of tissue blocks is expected to be faster than in temperate climates. Shen et al. have studied the effect of storage duration on K-*ras* gene detection in FFPE colorectal tissues and found that detection of K-*ras* decreased significantly after more than 2 years [53].

The major limitation of this study is the relatively small sample size for each age group and years of surgery. This is because surgically-resected appendixes that were histologically normal were not commonly encountered. With larger numbers, it may be possible to construct a working reference matrix. Notwithstanding, our comparison of TCRs between age-groups and also the comparison of TFIs between duration of tissue storage have revealed statistically relevant results. Additionally, measurement of a larger number of cells would be more ideal to counter variability in telomere length due to heterogeneity of cellular and tissue types. We have analyzed epithelial and stromal cells separately to reduce variability from tissue heterogeneity. Also as we were dealing with tissue sections and not smears, we had to contend with the problem of overlapping nuclei–which were excluded from the assessment. We also limited our selection to interphase cells with two identifiable centromeres (for study A) to reduce bias in favor of telomeres and to achieve a better standardization in the calculation of the TCR. Because of these strict inclusion criteria, the numbers of suitable nuclei available for this study are less than in other studies. Even so, with the analysis of fewer (but better quality) cells, our findings have shown statistically relevant results.

## Conclusions

This proof of concept study has shown that telomere lengths can be measured from FFPE tissues using Q-FISH, that telomere length varies with chronological age and that storage duration of FFPE tissues can introduce errors to telomere length measurements. Consideration of these confounding factors is important in studies where test groups being compared are not age-matched, and also in studies using FFPE tissues of variable storage duration. It has raised the notion that non-diseased tissues may be relevant as a tissue specific age-baseline reference in telomere studies. Since telomere length decreases with aging, determining an estimated telomere length at a certain age category, allows for factoring in of telomere attrition due to physiological age in studies on telomere length quantification in various pathological conditions, such as cancer. In addition, we have shown that FFPE tissues archived for more than 2 years are suboptimal for telomere Q-FISH analysis as TFI signals are significantly reduced. We suggest that for studies on telomere length quantification utilizing FFPE tissues, the blocks should be as recent as possible and preferably stored for less than 2 years.

## Supporting Information

S1 File Epithelial telomere length (TCR) values of each sample in study A. (XLSX)

S2 File Stromal telomere length (TCR) values of each sample in study A. (XLSX)

S3 File Telomere length (TFI) values of each sample in study B. (XLSX)

S4 File Telomere length (both TCR and TFI) values for both normal testicular tissues (controls). (XLSX)
