# Correction: Assessment of Telomere Length in Archived Formalin-Fixed, Paraffinized Human Tissue Is Confounded by Chronological Age and Storage Duration

**DOI:** 10.1371/journal.pone.0168238

**Published:** 2016-12-09

**Authors:** Po-Lian Kong, Lai-Meng Looi, Tze-Pheng Lau, Phaik-Leng Cheah

## Notice of Republication

This article was republished on September 27, 2016, due to the release of confidential or copyrighted material. Please download this article again to view the correct version.
